# Interaction between emotion lability, emotion regulation and structural brain abnormalities in patients with anorexia nervosa and borderline personality disorder

**DOI:** 10.3389/fnins.2025.1545599

**Published:** 2025-10-07

**Authors:** Magdalena Wayda-Zalewska, Agnieszka Pluta, Tomasz Wolak, Mateusz Wojtczak, Jakub Wojciechowski, Katarzyna Kucharska

**Affiliations:** ^1^Institute of Psychology, Cardinal Stefan Wyszyński University in Warsaw, Warsaw, Poland; ^2^Faculty of Psychology, University of Warsaw, Warsaw, Poland; ^3^World Hearing Center, Institute of Physiology and Pathology of Hearing, Kajetany, Poland

**Keywords:** anorexia nervosa, borderline personality disorders, emotion regulation, MRI, gray matter volume, brain structure, subcortical brain structures

## Abstract

**Objective:**

Emotion dysregulation is a significant challenge in borderline personality disorder (BPD) and anorexia nervosa (AN). These disorders often exhibit maladaptive emotion regulation (ER) strategies, which can vary widely. This study aimed to assess cortical thickness and volume, and their relationship with emotional lability and regulation in females with AN and BPD.

**Methods:**

The study involved 32 subjects with AN, 46 with BPD, and 41 age-matched healthy controls (HC). Participants underwent brain magnetic resonance imaging utilizing structural magnetic resonance imaging (MRI) alongside validated psychological assessment tools to measure emotional lability and regulation. Volumetric and cortical thickness measurements were assessed, and semi-partial Pearson’s correlations were used to analyze associations with psychological subscales.

**Results:**

Subjects with AN demonstrated higher level of adaptive strategies and lower maladaptive strategies compared to those with BPD, except in self-blame, which showed no difference between clinical groups. The results suggest that BPD patients demonstrate significantly higher emotional lability and reliance on maladaptive regulation strategies. Neuroimaging data revealed significant structural differences between HC and the clinical groups, particularly in the prefrontal cortex, nucleus accumbens, hippocampus, and insula. Few significant correlations were also observed between variables associated with emotion dysregulation/lability and gray matter volume/thickness in the frontal regions, cingulate cortex, insula, and basal nuclei.

**Conclusion:**

The clinical groups differed from each other and from the group of HC in terms of the variables studied. The study highlights the critical role of neurobiological mechanisms in informing the development of targeted therapeutic interventions for emotion dysregulation.

## Introduction

Patients suffering from borderline personality disorder (BPD) and anorexia nervosa (AN) present with a high-risk profile of self-injury vs. starvation, but also of suicide attempts (up to 20%) (Biskin, 2015; Franko and Keel, 2006). Various components of emotional lability and emotion dysregulation (such as abnormal patterns of emotion dynamics and maladaptive aspects of applying emotion regulation (ER) strategies) constitute some of the most serious difficulties in BPD (Stepp et al., 2014; Selby and Joiner, 2009) and in AN (Oldershaw et al., 2015; Engel et al., 2013; Lavender et al., 2013; Lavender et al., 2016; Goldschmidt et al., 2015; Seidel et al., 2016). Researchers suggest that affective states of AN individuals might be characterized by lability of negative and positive emotions (Lavender et al., 2013; Selby et al., 2014). Weight loss behaviors can be positively reinforced through positive emotions resulting from successful weight loss or self-control. The same behaviors can be negatively reinforced by mitigating negative emotions resulting from weight gain concerns. Evidence accumulated to date in a recent meta-analysis confirms the key role of emotional lability in the development and maintenance of BPD, for instance impaired processing of emotional and environmental stimuli (i.e., stressors) negatively mediates the behavior of BPD patients, with a strong impact on daily life functioning (D'Aurizio et al., 2023). Instability of emotional reactions is a diagnostic criterion for BPD in both the DSM-5 and the ICD-10 (Santangelo et al., 2014).

Currently, the state of knowledge on the specificity of emotion regulation in AN and BPD remains insufficient. Interestingly, in these disorders maladaptive ER can take the form of contrasting strategies ranging from self-injury (resulting from disinhibition) to emotion suppression manifested as, e.g., starvation (resulting from excessive self-control).

A theoretical model concerning the role of emotion regulation in the development and maintenance of AN is partially based upon the model proposed by the biosocial theory specifically to AN (Haynos and Fruzzetti, 2011). The model assumes a deficit of adaptive ER strategies and an excess of maladaptive ER strategies. When it comes to adaptive ER strategies, the authors point to a deficit of cognitive reappraisal. As far as maladaptive ER strategies are concerned, the authors highlight an excess of emotional avoidance, emotion suppression, and rumination (in the form of starvation as well as inhibition of one’s emotional expression).

These strategies are positively reinforced, because they result in a temporary decrease in negative emotions and thus constitute an important factor in the maintenance of the disorder (Kret and Ploeger, 2015; Fox, 2009). In individuals with AN, emotion suppression is more pronounced compared to other clinical groups and healthy individuals. It also acts as a mediator between anxiety, depression symptoms, and the severity of eating disorder symptoms (Wildes et al., 2010).

Linehan’s (1993) theory suggests that difficulties in emotion regulation contribute to BPD, which can stem from both biological factors and a dysfunctional upbringing. According to this theory, individuals with BPD struggle with regulating their emotions, leading to significant emotional challenges (Jazaieri et al., 2013). Further developments of this theory resulted in the creation of emotional cascade model, highlighting how dwelling on negative emotions can escalate into self-harming behaviors, which individuals with BPD often use to stabilize intense emotions (Selby and Joiner, 2009). Ruminating on negative emotions is a common maladaptive strategy in BPD (Smith et al., 2006), along with other strategies like suppression and avoidance (Daros et al., 2018). Blaming others is also prevalent in BPD and is rooted in the disorder’s core psychopathology as described in the DSM-5 (American Psychiatric Association, 2013).

In a nutshell, the studies on AN and BPD reveal three shared dimensions: emotion lability, emotion dysregulation, and emotion regulation. Exploring the interplay among these dimensions requires attention to the neurostructural correlates within these disorders. This approach offers insights into their mutual relationship, enriching our understanding of their underlying mechanisms.

### Structural brain changes in AN and BPD in regard to emotional lability and emotion regulation

Undoubtedly, structural brain abnormalities play a crucial role in understanding the complexities of AN and BPD, providing insights into both transdiagnostic and disorder-specific mechanisms underlying emotional lability and regulation (Tose et al., 2024; Giannoulis et al., 2025).

Neuroimaging studies conducted on individuals with AN indicate that malnutrition in anorexia significantly impacts brain structure, yet the precise influence of these changes on patients’ behavior remains uncertain (Frank et al., 2019). What is evident, however, is their contribution to the maintenance of a distorted body image, diminishing learning capacity, intensifying behavioral rigidity, and exacerbating cognitive distortions. However, there is a lack of studies on the potential relationship between brain morphometric changes and ER strategies among individuals with AN.

In regards to subjects with BPD, neuroimaging studies yielded structural and functional abnormalities in the frontal-limbic network, which includes regions involved in emotion processing (e.g., amygdala, insula, hippocampus) and frontal brain areas involved in regulatory control processes [e.g., anterior cingulate cortex (ACC), medial orbitofrontal cortex (mOFC), and dorsolateral prefrontal cortex (dlPFC)] (Tebartz van Elst et al., 2003; Krause-Utz et al., 2018; Driessen et al., 2000; Rüsch et al., 2003; Schmahl et al., 2003).

It is important to acknowledge that the relationship between emotional lability, emotion regulation, and structural brain changes in the MRI environment remains inadequately understood for both AN and BPD. The scarcity of neuroimaging research exploring these dimensions represents a significant gap in our understanding of the neurobiological mechanisms underlying these disorders, emphasizing the need for further scientific inquiry.

The aim of this study is to address the identified gaps by examining group differences in emotion lability, emotion regulation, and brain morphometry among individuals with AN and BPD compared to healthy controls as well as to explore the relationship between emotional lability, emotion dysregulation, and structural brain abnormalities in patients diagnosed with AN and BPD.

### Public significance statement

Our study has numerous practical implications that constitute a basis for improving therapeutic interventions in emotion dysregulation, and self-harm/suicide prevention programs for BPD and AN. The project has a translational value by enabling the translation of research into the clinical setting and thus promote evidence-based practice. Considering serious emotional difficulties that individuals with BPD and AN struggle with, conducting research in this domain appears highly socially beneficial.

## Methods

### Participants

This study is part of a larger study on emotion dynamics and emotion regulation in borderline personality disorder and anorexia nervosa. The study included data collected from February 2021 to March 2023.

A total of 119 subjects (*N* = 119) participated in the study. Due to the higher prevalence among women of AN diagnoses and unavailability of male patients treated in a clinical setting (90%) (American Psychiatric Association, 2013), the study included only female subjects. Patients with AN-*Binge Eating*/*Purging Type* were not recruited to the study due to differences in emotion regulation relative to the group of patients with AN *Restricting* Type (Danner et al., 2014). All subjects were at age between 18 and 45, polish nationality, Caucasian, students or working adults with an average socioeconomic status.

To be qualified for the first stage of the study, participants had to meet the following eligibility criteria: age 18–45, right-handedness, at least primary education, no diagnosed intellectual disability, verbal contact to understand the research protocol and complete all parts of the study, Polish as a native language, written informed consent to participate in the study, and signing the RODO. Disqualification criteria for all groups were as follows: serious somatic diseases, neurodevelopmental disorders, severe neurological dysfunction, previous brain injury, addiction to alcohol or another psychoactive substance in the past 6 months, contraindications to MRI – pregnancy, metal components in the body (such as: pacemaker, permanent braces [retention braces for lower jaw only is not an obstacle]), tattoos on the face or neck, permanent makeup, uncorrected visual impairment below −5 or above +5 diopters, claustrophobia (fear of cramped rooms). Disqualification criteria for healthy women were as follows: past or current mental disorder, current psychotherapy, BMI (Body Mass Index; body mass index) < 18.5 or ≥ 30. Disqualification criteria for clinical groups were as follows: antisocial personality disorder, schizophrenia spectrum disorder or other psychotic disorder, bipolar affective disorder, current episode of mania or hypomania. In addition, in the group with borderline personality disorder: eating disorder, BMI (body mass index) < 18.5 or ≥ 30 and in the group with anorexia: borderline personality disorder, BMI (body mass index) < 14 were additional disqualifying criteria for participation in the study.

Our aim was to recruit two homogenous clinical groups: patients with restrictive AN (type I) and individuals with BPD, diagnosed according to DSM-5 (American Psychiatric Association, 2013) psychiatric classification criteria and confirmed by a psychiatrist. In addition, all participants underwent the SCID-5 interview combined with the BPD checklist to allow detection of personality disorder symptoms. To ensure group homogeneity, particular effort was made to exclude AN patients with any comorbid personality disorder (including BPD). Individuals with a dual diagnosis (AN and BPD) or those not meeting the eligibility and exclusion criteria were not included in the study.

Subjects were divided into three groups: 32 right-handed women with a diagnosis of anorexia nervosa (ANg; mean age: 26.6 years, *SD* = 7.1; mean BMI: 16.3 Kg/m^2^, *SD* = 2.2; mean year of education = 15.3, *SD* = 2.6) restrictive subtype according to the DSM -V criteria (American Psychiatric Association, 2013), 46 subjects with Borderline Personality Disorder (BPDg; mean age: 26.0 years, *SD* = 5.4; mean BMI = 22.9 Kg/m^2^, *SD* = 3.6; mean year of education = 16.2, *SD* = 2.9), and 41 healthy right – handed women (HCg; mean age: 27.1 years, *SD* = 5.7; mean BMI = 21.9 Kg/m^2^, *SD* = 3.1; mean year of education = 17.4, *SD* = 2.5).

The groups differed significantly on BMI (*F* = 46.19; *p* < 0.001) – ANg had statistically lower BMI than both HCg and subjects with BPD (*p* < 0.05) and on number of years of education (*F* = 38.61; *p* = 0.007) – subjects with AN had significantly fewer years of education than HCg (*p* = 0.005). The groups did not differ on age (*F* = 0.40; *p* = 0.675).

ANg and BPDg were recruited from therapeutic wards and psychiatric outpatient clinics in private and public medical facilities in Poland. The HCg were recruited voluntarily by means of flyers posted on social media.

All participants provided written informed consent to participate in the study prepared in accordance with the current version of the Helsinki Declaration, as well as a statement of familiarity with information on the processing of personal data – General Data Protection Regulation [Regulation (EU) 2016/679 (GDPR)]. The study was conducted in accordance with the ethical standards of UKSW (no. KEiB-05/2020).

### Measures

Subjects were asked to complete self-report scales measuring clinical constructs (BPD Checklist, EAT-26, and HADS) and went through personality disorders (PDs) assessment with the SCID-5-PD. In Part I, participants were asked to fill in self-report scales on various components of emotion dysregulation (EDS, CERQ, RESS-24, ALS-18). In Part II, subjects underwent a neuroimaging procedure.

#### Self-report scales

Emotion Dysregulation Scale, short version (ED – Scale short; Kröger et al., 2011; Polish validation: Kucharska et al., in preparation) was used to assess the severity of emotion dysregulation (reliability: HC – *α* = 0.91, ANg – α = 0.92, BPDg – α = 0.92).

Cognitive Emotion Regulation Questionnaire (CERQ, Garnefski et al., 2002; Polish validation: Marszał-Wiśniewska and Fajkowska, 2010) was used to identify the cognitive emotion regulation strategies after having experienced negative events or situations (reliability: HCg – *α* = 0.58–0.87, ANg – α = 0.49–0.94, BPD – α = 0.52–0.90).

The Regulation of Emotion Systems Survey (RESS-24, De France and Hollenstein, 2017; Polish validation: Kucharska et al., in preparation) was used to capture six common emotion regulation strategies: *Distraction, Rumination, Reappraisal, Suppression, Engagement, Arousal Control* (reliability: HCg – *α* = 0.76–0.95, ANg – α = 0.74–0.95, BPDg – α = 0.65–0.91).

The Affective Lability Scale (ALS-18, Oliver and Simons, 2004, Polish validation by Kucharska et al., in preparation) was used to measure affective lability (reliability: HCg subscales α = 0.87–0.89 and total score α = 0.94, ANg – subscales α = 0.88–0.92 and total score 0.95, BPD – subscales α = 0.79–0.87 and the total score α = 0.92).

Eating Attitudes Test (EAT-26, Garner et al., 1982; Polish adaptation: Rogoza et al., 2016) was used to identify eating disorder risk based on attitudes, feelings and behaviors related to eating (reliability: HCg – α = 0.58, ANg – α = 0.94, BPDg – α = 0.90).

The Hospital Anxiety and Depression Scale (HADS, Hospital Anxiety and Depression Scale) tool (Zigmond and Snaith, 1983; Polish validation: Nezlek et al., 2019) was used to assess the severity of anxiety and depression (reliability: HCg α = 0.82 for the anxiety scale and α = 0.71 for the depression scale, ANg α = 0.85 for the anxiety scale and α = 0.81 for the depression scale, BPDg: α = 0.82 for the anxiety scale and 0.78 for the depression scale).

### MRI data acquisition

The T1-weighted structural images were collected at Biomedical Research Centre, World Hearing Center, Kajetany, Poland on a 3 T Siemens Prisma scanner equipped with a 64-channel head coil. Structural T1-weighted MRI images were acquired with TR = 2,400 ms, TE = 2.74 ms, flip angle = 8°, FOV = 256 mm, matrix 320×320, 240 slices, and with 0.8 mm isotropic voxels, Acquisition time 6:52 min.

#### Structural morphometry analysis

Volumetric and cortical thickness measurements were obtained with FreeSurfer 7.3 (Fischl et al., 2004)^1^. The analysis was carried out with a standard (a) surface-based stream: volume registration with the MNI305 (Collins et al., 1994), intensity normalization, skull stripping, segmentation, tessellation of the gray matter–white matter boundary, automated topology correction, and surface deformation following intensity gradients; and (b) a volume-based stream: volume registration with the MNI305, initial volumetric labeling, intensity normalization, a high dimensional nonlinear volumetric alignment to the MNI305, after the preprocessing the volumes were labeled again. Image quality was assessed by visual inspection to assure the results met quality assurance standards for both cortical and subcortical segmentation. For analysis we used Desterieux atlas for cortical regions and anatomical segmentation atlas for subcortical regions.

### Statistical analysis

**Figure 1:**
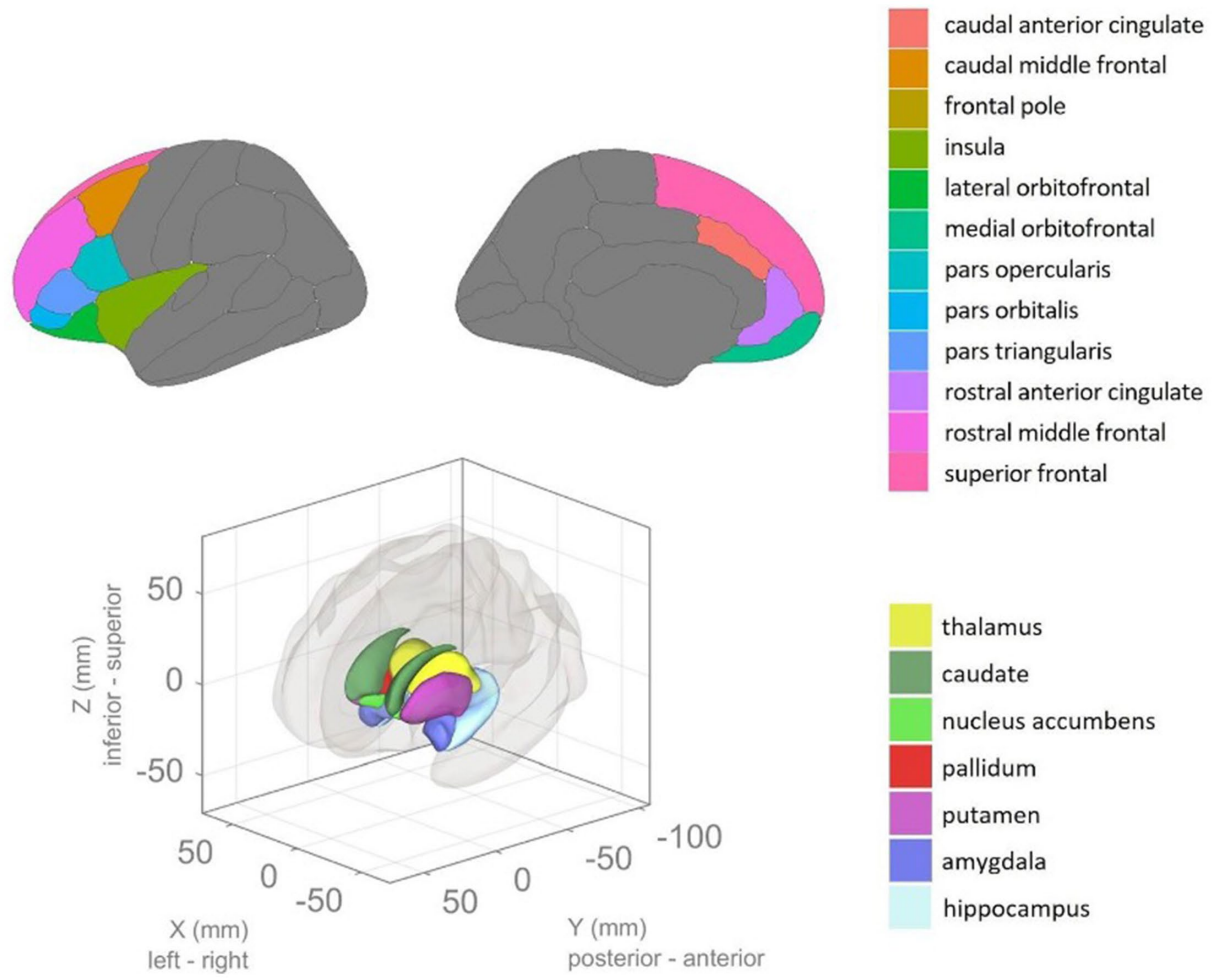
Areas of interest highlighted in color were used for morphometric analysis. The top panel highlights cortical areas, while the bottom panel highlights subcortical areas. The color of each region represents areas from the Desterieux atlas or the anatomical segmentation atlas, respectively. Anatomical region names annotated on the legend.

Data was tested for normality of distribution and homogeneity of variance using the Kolmogorov–Smirnov test and Levene’s Test (respectively). Based on these results, parametric tests were applied. Demographic, psychological, and clinical variables were subjected to statistical analysis with ANOVA. Regions of interest (ROIs) were analysed using ANCOVA including total intracranial volume (TIV, obtained from FreeSurfer) as a covariate. The cortical thickness is reported in mm (milimeters) and cortical volume is reported in cubic milimetres (mm3). All ROIs included in the analyses are visualized in Figure 1.

For all statistical analyses a *p* value < 0.05 (two-sided) was considered as statistically significant. All the post-hoc tests were adjusted for multiple comparisons using Bonferroni correction (see Table 1).

**Table 1:** Results of ANCOVA of gray matter volume and thickness between BPD, AN and HC groups.

Brain region	*F*	*p*	ηg^2^	*Post-hoc* Bonferroni	HC		AN		BPD	
					M	SE	M	SE	M	SE
Regional gray matter thickness (mm)										
Accumbens LH	6.642	0.002	0.104	HC, AN>BPD	511.35	14.05	503.20	16.32	445.65	13.39
Accumbens RH	5.981	0.003	0.094	HC>BPD	623.64	14.92	595.29	17.34	553.02	14.23
Pars opercularis LH	4.59	0.012	0.07	HC>AN	4932.91	101.44	4489.48	117.87	4619.49	96.73
Hippocampus RH	3.453	0.035	0.057	HC>BPD	4241.03	48.41	4181.73	56.25	4068.33	46.16
Regional gray matter volume (mm^3^)										
Insula LH	4.932	0.009	0.079	AN>BPD	3.07	0.02	3.13	0.02	3.04	0.02
Frontal pole RH	4.952	0.009	0.079	AN<BPD	2.71	0.04	2.58	0.05	2.77	0.04
Rostral middle frontal LH	3.42	0.036	0.056	BPD > AN	2.39	0.02	2.33	0.02	2.40	0.02

*p < 0.05. **p < 0.01. ***p < 0.001. AN, Anorexia nervosa; BPD, Borderline personality disorder; HC, Healthy controls; LH, Left hemisphere; RH, Right hemisphere; F, F value; ηg^2^, Generalized eta squared; M, Mean; SE, Standard error.

To further investigate the clinical meaning of regional volume and thickness, we run semi-partial Pearson’s correlations in BPD group and AN group (separately) between TIV-adjusted morphometric characteristics and clinical measurements of emotional lability and dysregulation. The Benjamini-Yekutieli (FDR) correction was applied for multiple comparisons (see Tables 2, 3). All statistical analyses were carried out using R software (v4.1.0; R Core Team, 2021).

**Table 2:** Semi-partial correlations between clinical measurements and regional gray matter volumes or thickness with uncorrected and FDR adjusted *p*-values in AN group.

Correlation pairings		*r*	*p*_raw_	*p*_adj_fdr_
Regional gray matter thickness				
ALS total score	Superior frontal RH	0.400	0.026	0.757
CERQ_POSREAPP	Frontal pole LH	−0.381	0.035	0.806
CERQ_POSREAPP	Superior frontal LH	−0.366	0.043	0.806
CERQ self-blame	Caudal anterior cingulate LH	−0.511	0.003	0.281
CERQ self-blame	Rostral anterior cingulate RH	−0.397	0.027	0.757
CERQ self-blame	Medial orbitofrontal LH	−0.373	0.039	0.806
RESS supression	Rostral anterior cingulate LH	−0.512	0.003	0.281
RESS supression	Medial orbitofrontal RH	−0.477	0.007	0.376
RESS supression	Medial orbitofrontal LH	−0.456	0.010	0.414
Regional gray matter volume				
ALS total score	Pallidum LH	0.493	0.005	0.750
ALS total score	Pallidum RH	0.484	0.006	0.750
ALS total score	Superior frontal LH	0.360	0.047	0.777
EDS score	Lateral orbitofrontal RH	−0.377	0.037	0.777
EDS score	Pallidum RH	0.366	0.043	0.777
EDS score	Pallidum LH	0.358	0.048	0.777
CERQ catastrophizing	Pars opercularis RH	−0.403	0.025	0.777
CERQ catastrophizing	Lateral orbitofrontal RH	−0.383	0.033	0.777
CERQ self-blame	Lateral orbitofrontal RH	−0.408	0.023	0.777

LH, Left hemisphere; RH, Right hemisphere; *r*, Pearson’s correlation coefficient.

**Table 3:** Semi-partial correlations between clinical measurements and regional gray matter volumes or thickness with uncorrected and FDR adjusted *p*-values in BPD group.

Correlation pairings		*r*	*p*_raw_	*p*_adj_fdr_
Regional gray matter thickness				
ALS score	Rostral middle frontal RH	−0.445	0.002	0.123
ALS score	Pars orbitalis RH	−0.428	0.003	0.141
ALS score	Medial orbitofrontal LH	−0.407	0.005	0.178
ALS score	Pars triangularis RH	−0.394	0.007	0.178
ALS score	Rostral middle frontal LH	−0.388	0.008	0.178
ALS score	Medial orbitofrontal RH	−0.363	0.014	0.265
ALS score	Superior frontal LH	−0.350	0.019	0.267
ALS score	Caudal anterior cingulate RH	−0.307	0.040	0.448
EDS score	Insula RH	0.344	0.021	0.268
EDS score	Caudal anterior cingulate LH	−0.320	0.032	0.387
CERQ catastrophizing	Insula RH	0.503	0.000	0.037
CERQ catastrophizing	Frontal pole RH	0.502	0.000	0.037
CERQ_POSREAPP	Frontal pole RH	−0.358	0.016	0.266
CERQ rumination	Insula LH	0.391	0.008	0.178
CERQ rumination	Frontal pole RH	0.348	0.019	0.267
CERQ rumination	Insula RH	0.300	0.045	0.472
CERQ rumination	Caudal middle frontal RH	0.282	0.061	0.507
Correlation pairings	*r*	*p*_raw_	*p*_adj_fdr_	0.482
CERQ self-blame	Frontal pole RH	0.292	0.052	0.482
Regional gray matter volume				
ALS score	Caudal middle frontal RH	0.372	0.012	0.793
ALS score	Pars triangularis RH	−0.317	0.034	0.793
CERQ self-blame	Parsopercularis LH	−0.359	0.015	0.793
CERQ self-blame	Superior frontal RH	−0.344	0.021	0.793
CERQ self-blame	Insula LH	−0.311	0.037	0.793
CERQ self-blame	Pars triangularis RH	−0.303	0.043	0.793

LH, Left hemisphere; RH, Right hemisphere; *r*, Pearson’s correlation coefficient.

### Transparency and openness

We report that all measures in the study follows JARS (Appelbaum et al., 2018). This study’s design and its analysis were not pre-registered.

## Results

**Figure 2:**
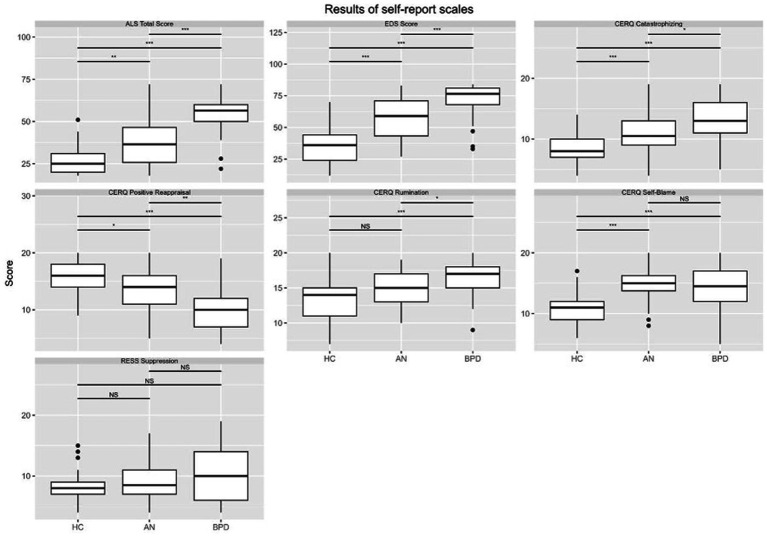
Emotion regulation strategies.

The findings presented in Figure 2 illustrate the outcomes derived from intergroup comparisons concerning variables associated with emotional lability, emotion dysregulation, and emotion regulation strategies across all study groups. The results reveal statistically significant differences between the healthy and clinical groups, as well as among the clinical groups themselves.

In general, healthy controls had statistically lower scores in maladaptive ER strategies and significantly higher scores in adaptive ER strategies in comparison to subjects from clinical groups.

In particular, healthy controls yielded lower scores on subscales capturing catastrophizing (in comparison to both clinical groups) (*F* = 23.51, *p* < 0.001, partial *n*^2^ = 0.29), rumination (in comparison to both clinical groups) (*F* = 14.21, *p* < 0.001, partial *n*^2^ = 0.20), self – blame (in comparison to both clinical groups) (*F* = 19.61, *p* < 0.001, partial *n*^2^ = 0.25). Furthermore, they obtained significantly higher scores in adaptive strategy such as Positive Reappraisal (*F* = 23.94, *p* < 0.001, partial *n*^2^ = 0.29) in comparison to both clinical groups. No significant differences were observed between the study groups for the emotion suppression variable.

Furthermore, subsequent analyses examined intergroup differences in emotional lability (*F* = 73.75, *p* < 0.001, partial *n*^2^ = 0.56) and general emotion dysregulation (*F* = 80.46, *p* < 0.001, partial *n*^2^ = 0.58). The clinical groups differed from the healthy group in terms of lability on all subscales and the total score and also in terms of emotion dysregulation. The BPD group had the highest scores for emotional lability and overall emotion dysregulation compared to with AN and HC groups. The AN group differed significantly from the HC group on these variables.

With regards to morphometric measurements, we observed group differences in the volume- or thickness-based measures of nucleus accumbens (NAc) (bilaterally), left pars opercularis and right hippocampus, left insula, right frontal pole and left rostral middle frontal gyrus. Further post-hoc analysis revealed that subjects with BPD exhibited smaller volume- or thickness-based measures in the NAc bilaterally (*F* = 6.64, *p <* 0.002, partial *n*^2^ = 0.104; *F* = 5.98, *p <* 0.003, partial *n*^2^ = 0.094) and right hippocampus compared to HC (*F* = 3.45, *p <* 0.035, partial *n*^2^ = 0.057). BPD group also showed decreased volume in the left NAc and left insula (*F* = 4.93, *p <* 0.009, partial *n*^2^ = 0.079) compared to individuals with AN. Subjects with AN displayed decreased volume or thickness-based measures in the pars opercularis in comparison to HC (*F* = 4.59, *p <* 0.012, partial *n*^2^ = 0.07), and in frontal pole (*F* = 4.95, *p <* 0.009, partial *n*^2^ = 0.079) as well as rostral middle frontal gyrus (*F* = 3.42, *p <* 0.036, partial *n*^2^ = 0.056) in comparison to subjects with BPD. Group comparisons of volume- and thickness-based measures are presented in Table 1.

### Results for correlation pairings in AN group

Although several comparisons of clinical measurements and regional brain volume/thickness of gray matter reached nominal statistical significance at *p* < 0.05 prior to adjustment for multiple testing, application of the false discovery rate (FDR) correction substantially attenuated these associations. After controlling for the FDR at the 5% level, the majority of these findings did not remain statistically significant (*p.adj.fdr* > 0.05), suggesting that they may represent false positives and should be interpreted with caution.

In AN group self-blame strategy correlated negatively with right rostral (*r* = −0.40; *p* < 0.027; q), left medial (*r* = −0.37; *p* < 0.039) and left caudal (*r* = −0.51; *p* < 0.003) anterior cingulate gyrus thickness and right lateral orbitofrontal (*r* = −0.41; *p* < 0.023) volume. Suppression correlated negatively with left rostral anterior cingulate gyrus (*r* = −0.51; *p* < 0.003) and medial anterior cingulate (LH and RH) thickness (*r* = −0.48; *p* < 0.007 and *r* = −0.46; *p* < 0.010). Positive reappraisal correlated negatively with the left frontal pole (*r* = −0.38; *p* < 0.035) and left superior frontal gyrus (SFG) (*r* = −0.37; *p* < 0.043) thickness. Right SFG thickness (*r* = 0.40; *p* < 0.026) and left SFG volume (*r* = 0.36; *p* < 0.047) as well as pallidum volume (left and right) (*r* = 0.49; *p* < 0.005 and *r* = 0.48; *p* < 0.006) correlated positively with emotional lability.

An interesting finding was made regarding general emotion dysregulation: this psychological construct correlated positively with pallidum volume (left and right) (*r* = 0.37; *p* < 0.043 and *r* = 0.36; *p* < 0.048) and negatively with right lateral orbitofrontal gyrus volume (*r* = −0.38; *p* < 0.037).

Results are presented in Table 2.

### Results for correlation pairings in BPD group

For BPD group there were found more significant correlations for ER and brain structures comparing to AN group. In BPD group self-blame strategy correlated negatively with left insula (*r* = −0.0.31; *p* < 0.037), left pars opercularis (*r* = −0.36; *p* < 0.015), right pars triangularis (*r* = −0.30, *p* < 0.043), right SFG (*r* = −0.34, *p* < 0.021) and positively with the right frontal pole thickness (*r* = 0.29; *p* < 0.052). There were no found any significant correlations with supression. Positive reappraisal correlated similarly to AN group negatively in the frontal pole but in the right hemisphere (*r* = −0.36; *p* < 0.016). Rumination correlated positively with insula (left and right; *r* = 0.39; *p* < 0.008 and *r* = 0.30; *p* < 0.045), frontal pole (right) (*r* = 0.35; *p* < 0.019) and caudal middle frontal (right) (*r* = 0.28; *p* < 0.061). Emotional lability correlated negatively with thickness and volume of several brain structures such as: rostral middle frontal gyrus (left and right) (*r* = −0.39; *p* < 0.008 and *r* = −0.45; *p* < 0.002), left SFG (*r* = −0.35; *p* < 0.019), right pars orbitalis (*r* = −0.43; *p* < 0.003), right pars triangularis (*r* = −0.39; *p* < 0.007), left medial orbitofrontal (*r* = −0.41; *p* < 0.005), right caudal anterior cingulate (*r* = −0.31; *p* < 0.040) and positively with right caudal middle frontal gyrus (*r* = 0.37; *p* < 0.012). General emotion dysregulation correlated positively with right insula (*r* = 0.34; *p* < 0.021) and negatively with caudal anterior cingulate thickness (*r* = −0.32; *p* < 0.032).

What needs to be underlined, there’ve been only two results still *p* < 0.05 when use the FDR-adjusted *p*-values. There was a positive correlation between catastrophizing and right insula (*r* = 0.50; *p* < 0.001) and between catastrophizing and frontal pole thickness (*r* = 0.50; *p* < 0.001). Results are presented in Table 3.

## Discussion

This study aimed to evaluate emotional lability and emotion regulation in AN and BPD, as well as to investigate potential associations between these variables and morphometry of brain structures. The goal was to differentiate between the two disorders and uncover shared structural correlates across both clinical groups.

### Emotion lability

In the present study, BPD patients were found to be more emotionally labile and with overall greater emotion dysregulation than the other groups and had greater saturation of these traits than the group with anorexia. The findings presented in our study align with existing research literature (Miller et al., 2022; Voderholzer et al., 2021). Emotional lability and dysregulation manifest differently in individuals with AN compared to those with BPD. Individuals with BPD may display more pronounced outward symptoms such as impulsive behaviors, acting out, Non-Suicidal Self-Injuries (NSSI), or suicide threats, which serve to stabilize emotions in this disorder (Vansteelandt et al., 2017). In the case of people with AN, their difficulty in experiencing emotions and their volatility takes a more self-aggressive and intrapsychic form and may be masked externally, for example, in order to maintain a good image, shame or because of experienced guilt (Panero et al., 2022). In the context of people with AN, researchers have shown that high levels of both positive and negative emotional lability, and the interaction between the two, were associated with more frequent eating-related illness behaviors (Selby et al., 2014).

### Emotion regulation

Healthy individuals generally demonstrate a higher propensity for employing adaptive emotion regulation strategies, such as positive reappraisal, compared to both clinical groups. Moreover, healthy subjects exhibit a reduced frequency of non-adaptive strategies relative to clinical groups, potentially attributable to enhanced, heightened emotional awareness, and a more integrated personality.

At the same time, patients with AN showed significantly higher levels of maladaptive emotion regulation strategies, including catastrophizing, rumination, and self-blame, compared to healthy controls. These findings are consistent with prior research indicating elevated levels of catastrophizing (Sternheim et al., 2012) and rumination (Startup et al., 2013) among individuals with AN compared to healthy individuals. In the ANg we observed lower inclination toward utilizing maladaptive strategies like rumination and catastrophizing compared to those with BPD, although the exception was self-blame, which was not significantly different in the two groups. This aligns with the prevailing psychopathological profile of individuals with anorexia, characterized by feelings of guilt, shame, and anger (Peterson and Fuller, 2019).

In the BPD group, our study revealed a greater propensity for employing maladaptive emotion regulation strategies such as rumination, self-blame, and catastrophizing compared to healthy controls. These findings are consistent with other research, where participants with elevated BPD traits showed significantly higher levels of self-blame (Law and Chapman, 2015). Researchers have also shown that catastrophizing is significantly associated with BPD symptoms (Selby and Joiner, 2009). These findings correspond with those of other studies that link high levels of ruminations in BPD to instability in interpersonal relationships, unstable self-image, self-injury, and impulsivity (Richman Czégel et al., 2022).

### Structural brain changes

In our analysis of volumetric and morphometric measurements, we observed several noteworthy group differences that partially align with prior research findings. These differences offer insights into the neural mechanisms underlying the clinical symptoms exhibited by individuals with AN and BPD.

An interesting area that differentiates the study groups is NAc, a region within the reward circuitry implicated in assigning subjective value to both positive and negative stimuli. Our findings reveal that the BPD group is characterized by a smaller bilateral NAc than HC, and a smaller left NAc relative to AN. The above results cannot be directly related to other reports due to limited structural MRI study results on NAc in the BDP group. Only two fMRI studies in BPD patients show reduced activity of the NAc relative to the HC on signals predicting an upcoming reward (Enzi et al., 2013; Herbort et al., 2016). The decreased volume of NAc in BPD may be explained by the altered action of the endogenous opioid system in the paraventricular nucleus in people with BPD (Prossin et al., 2010), which could potentially cause a change in the structure of dendritic branching and the number of dendritic spikes on medium spiny neurons in the NAc shell. The endogenous opioid system is implicated in BPD, particulary in its modulation of pain perception during self-injurious behavior and dissociative episodes (ibid.). Furthermore, this system plays a crucial role in regulating emotions and stress responses, often through impulsive and rewarding behaviors.

Moreover, our study showed no structural differences in bilateral NA in the AN group compared to HC, which remains consistent with some other studies (Frank et al., 2013; Leppanen et al., 2020). However, other findings were shown by King et al. (2015) indicating that individuals in the acute phase of anorexia had reduced NAc (King et al., 2015) compared to HC. This result may be explained in relation to the low BMI index (mean 14.8) and severely malnourished state of AN patients in the acute state of illness.

Our structural analyses revealed that the volume of the right hippocampus is reduced in the BPD group compared to the HC group, consistent with findings from previous studies (Depping et al., 2016).

We found that the left insula cortical thickness is greater in the AN group compared to BPD group, while both clinical groups did not significantly differ from the HC group. This aligns with a prior study where the thickness of the left and right insula cortex did not significantly differ between BPD and HC (Bruehl et al., 2013). The thicker insula cortex in the AN group compared to BPD patients may suggest differences in emotion processing between the two groups. The left insula is linked to parasympathetic activity and positive affect processing, while the right insula is associated with sympathetic activity and anxiety processing (Mortensen et al., 2016). The insula plays a crucial role in eating-related processes due to its involvement in taste processing, interoceptive awareness, and the motivational value of food, as well as its connections to the striatum, which regulates motivated/reward-related behavior (Nunn et al., 2011; Kerr et al., 2016; Scaife et al., 2016). Therefore, variations in cortical thickness in the left insula of the AN group may influence reward processing and group-specific perceptions of satiety (Frank et al., 2012). Conversely, in BPD, differences in insular cortex thickness may be related to the fact that the insular cortex may be more closely associated with parts of the medial circuit processing emotional aspects of pain (Datta and Arnsten, 2019).

In our study, we found an increased thickness of the right frontal cortex gray matter in the BPD group compared to AN, with no differences observed compared to the HC group, which aligns partially with previous findings indicating no distinctions between individuals with AN and HC (Myrvang et al., 2021). The role of the frontal pole, the largest cytoarchitectonic area of the human cerebral cortex, in clinical symptomatology remains uncertain. However, recent neuroimaging research has highlighted the relevance of the frontal pole, particularly Brodmann area 10 (BA10) known as the metacognitive hub, in the pathophysiology of borderline personality disorder (BPD) and anorexia nervosa (AN). BA10 is implicated in metacognitive functions such as self-monitoring, future-oriented thinking, and mentalizing—capacities that are often disrupted in both disorders. In BPD, hypoactivity or dysconnectivity in BA10 may contribute to unstable self-concept and impaired emotional insight, exacerbating impulsivity and interpersonal dysregulation (Khosravi, 2020; Wayda-Zalewska et al., 2021). Conversely, in AN, hyperactivation of BA10 may reflect excessive self-focus and cognitive rigidity, reinforcing maladaptive beliefs about body image and perfectionism. These divergent patterns suggest that BA10 dysfunction plays a transdiagnostic yet context-specific role in emotion regulation. Importantly, antidepressant treatment may modulate BA10 activity by enhancing prefrontal-limbic connectivity and supporting neuroplasticity, potentially improving metacognitive capacity and therapeutic engagement. Future studies should explore BA10 as a target for interventions aimed at restoring adaptive self-related processing in these populations.

In the AN group, we showed a smaller volume of gray matter in the left inferior frontal gyrus pars opercularis compared to HC. The pars opercularis, as a structure of the reflex system, has been repeatedly associated with inhibitory control. Other studies (Brodrick et al., 2021) also showed a smaller cortical thickness in the pars opercularis in the female AN group, but in the right cerebral hemisphere.

We showed that the thickness of the left rostral middle frontal was greater in the BPD group than in AN, but the two clinical groups were not significantly different from HCs. Other studies in the AN and BPD groups similarly showed no difference in rostral middle frontal thickness compared to HCs (Bruehl et al., 2013; Myrvang et al., 2021).

The observed brain structural alterations may be partly attributable to neurobiological deficits documented in other psychiatric conditions. Previous research has demonstrated reduced brain-derived neurotrophic factor (BDNF) levels in BPD (Forte et al., 2023), as well as in both unipolar and bipolar depression and in eating disorders such as AN (Monteleone et al., 2005, 2008). This suggests a potential overlap in the mechanisms underlying neuroplasticity and emotion dysregulation across these disorders. Chronic stress and nutritional deficiencies may further suppress BDNF synthesis, precipitating dendritic atrophy and gray matter reductions (Palamarchuk et al., 2023; Cao et al., 2024). Such neuroanatomical changes can undermine emotion regulation and help explain the pronounced emotional lability and maladaptive coping strategies observed in individuals with AN and BPD.

### Correlation between emotion lability, ER and brain structures

By analyzing the relationships between cortical thickness and behavioral variables, we noted some specific brain areas that correlated with at least two measured behavioral variables in the clinical groups studied. However, limited size of the groups may have affected the correlation results obtained; after applying corrections for multiple comparisons most of analysis become insignificant, which in turn forces us to consider the results as significant only at the level of trends.

In the case of BPD, the structures that were most frequently associated with both general emotion dysregulation and particular strategies were the insula (positive association with emotional lability and negative emotion regulation strategies such as: catastrophising, rumination, suppression, self-blame) and frontal pole (positive association with catastrophising, rumination, self-blame, and negative association with positive reappraisal). In contrast, in the AN group, such characteristic structures were medial orbitofrontal and rostral anterior cingulate (negative association with self-blame, suppression). The above associations show us that both BPD and AN may likely involve separate brain structures in processes related to emotion dysregulation and the use of analogous regulatory strategies.

### Clinical implications

The findings of this study can inform the development of targeted interventions and suggest two complementary avenues for enhancing emotion regulation in BPD and AN. First, therapeutic approaches should be tailored to each disorder’s specific profile: in BPD—where maladaptive strategies such as rumination and catastrophizing predominate alongside marked emotional lability—clinicians ought to emphasize cognitive restructuring and impulse-control techniques; in contrast, AN patients, despite generally relying more on adaptive strategies, often struggle with self-blame, indicating the utility of reinforcing self-compassion and cognitive reappraisal exercises. Second, the identified structural alterations point to promising neuromodulation targets: interventions directed at the prefrontal cortex such as Transcranial Magnetic Stimulation (TMS) may bolster top-down regulatory control over dysregulated emotional responses in both AN and BPD, potentially restoring more adaptive neural functioning. Other neuromodulation technique such as Neurofeedback training may be helpful in regulating brain activity by providing real-time feedback on neural responses, improving self-regulation. Future clinical studies may also consider the moderating role of temperament in those psychiatric disorders and their clinical dimensions such as psychopathology, suicide risk and treatment adherence (Favaretto et al., 2024).

### Limitations

First, our sample groups could be larger and performed on both female and male participants. Another limitation is the effect of the pharmacotherapy used by the subjects on their results, especially in terms of the severity of traits typical of the disorder, but also because of emotion regulation and emotional lability.

There is also uncertainty as to whether we can consider the results obtained as trait-related or state-related, especially in the case of the anorexia group, where the observed structural changes in the brain may be due to malnutrition. Acute AN is known to induce widespread gray and white matter with weight restoration, suggesting a predominately state-related pattern and conversely – some structural changes may persist post-recovery, hinting at trait-related features, but cross-sectional study like our cannot disentangle these mechanisms (Göller et al., 2022).

Although the results obtained might indicate which brain structures are neurocorrelates of emotion regulation and lability, determining the functional connectivity between these structures would allow for a more precise definition and understanding of the neural mechanisms and interrelationships associated with these psychological constructs. Conclusions based on correlation results should be drawn with caution as we cannot determine the causal role of any of the analyzed factors.

### Constraints on generality

Undoubted limitations related to generalization are the young age and gender of the subjects and also their relatively high socioeconomic status. In our study, we also did not differentiate between biological sex and social sex and did not consider the gender identification of the subjects or any other relevant demographics.

## Conclusion

Assessing interaction between emotion lability, emotion regulation and structural brain abnormalities in patients with AN and BPD appeared innovative and interdisciplinary based on current state of knowledge in this domain. The strength of this study was to have both clinical groups included and several maladaptive ER strategies analyzed that seem quite specific to either BPD or to AN. The results obtained confirm the data available in the literature and also clearly show us the directions of relationships. Moreover, this is the first study to analyze so many structures simultaneously in the context of these patient groups.

## Data Availability

The raw data supporting the conclusions of this article will be made available by the authors, without undue reservation.

## Footnotes

^1^ http://surfer.nmr.mgh.harvard.edu/
